# Nickel–Cobalt Bimetal Hierarchical Hollow Nanosheets for Efficient Oxygen Evolution in Seawater

**DOI:** 10.3390/ma17102298

**Published:** 2024-05-13

**Authors:** Rongzheng An, Guoling Li, Zhiliang Liu

**Affiliations:** 1 Institute of Materials for Energy and Environment, College of Materials Science and Engineering, Qingdao University, Qingdao 266071, China; anrongzheng1999@163.com; 2 College of Material Sciences and Chemical Engineering, Harbin Engineering University, Harbin 150001, China

**Keywords:** oxygen evolution reaction (OER), ultrathin nanosheets, hollow structure, electrolytic seawater, overall water splitting, sunlight driven

## Abstract

The electrochemical splitting of seawater is promising but also challenging for sustainable hydrogen gas production. Herein, ZIF-67 nanosheets are grown on nickel foam and then etched by Ni^2+^ in situ to obtain a hierarchical hollow nanosheets structure, which demonstrates outstanding OER performance in alkaline seawater (355 mV at 100 mA cm^−2^). Diven by a silicon solar panel, an overall electrolysis energy efficiency of 62% is achieved at a high current of 100 mA cm^−2^ in seawater electrolytes. This work provides a new design route for improving the catalytic activity of metal organic framework materials.

## 1. Introduction

Hydrogen energy, as a renewable and sustainable green energy source, has become the focus of global attention. Combined with vast marine resources, utilizing seawater to produce hydrogen is a highly encouraging renewable hydrogen production solution with great research and development prospects [1,2]. In the process of water splitting, the oxygen evolution reaction (OER) is the main determining step of energy conversion efficiency [3]. However, in natural seawater, the chlorine evolution reaction (CER) can be initiated at the anode, which requires the OER catalyst to reach a high current density output below 480 mV overpotential to prevent hypochlorite formation [4]. Therefore, the advancement of affordable, efficient, and corrosion-resistant catalysts holds significance for the promotion of seawater hydrogen production technology.

Transition metal organic framework material (TMOF), represented by three-dimensional (3D) porous material, is famous for its rich metal centers, microporous structures, and its variety [5,6]. TMOFs demonstrate broad application prospects in catalysis, separation, and energy storage [7,8,9]. Inside, the Co-based zeolitic imidazolate framework-67 (ZIF-67) is highly resistant to corrosion, possessing excellent electrical conductivity and strong mechanical properties, making it a highly promising option for electrolytic seawater splitting [10]. However, the catalytic activity of these materials remains to be improved. Co is the primary catalytically active site in the OER [11,12]. Hence, a viable approach to enhance the catalytic efficiency of Co-based ZIF-67 is to maximize the exposure of metal active sites. Layered double hydroxide (LDH), comprising 2D ultrathin nanosheets, is a reliable option for this objective. It has an ultrahigh surface area and facile synthesis methods, making it a viable option for imparting the electrocatalyst with the benefits of extensive surface areas and making it cost effective for enhancing its catalytic capabilities [13]. Recently, various LDH nanomaterials derived from ZIF-67, which was used as self-sacrificial template, have been widely studied. Xu et al. synthesized NiFeLDH with a hierarchical structure by incorporating nickel nitrate and ferric nitrate using ZIF-67 as a template [14]. Du and colleagues reported a method consisting of the in situ growth of ZIF-67 on a carbon cloth followed by the preparation of CoFe-LDH nanosheets, which significantly improved the catalytic performance of the OER [15]. This part of the research mainly focuses on constructing heterojunction interfaces, ignoring the changes in microstructure, which limits the sufficient exposure of active sites.

Herein, through an effective and flexible strategy, we synthesized NiCoLDH nanosheets on Ni foam (NiCoLDH/NF, NF means Ni foam) with a hierarchical hollow nanostructure using ZIF-67 as a template. Ultrathin nanosheets and hollow structures benefit from exposure to abundant electrocatalytic active sites. Ni^2+^ incorporation modulates the valence state of Co and optimizes the local electronic structure of the active centra. Thanks to the hierarchical hollow structure and optimized metal active sites, NiCoLDH/NF displays high catalytic activity and long stability (100 h) of the OER in alkaline seawater electrolyte. The evolution of the catalyst interface structure in simulated seawater was investigated, and the actual catalytically active sites were examined using in situ Raman spectroscopy. The current density of the dual-electrode electrolytic cell can reach 100.4 mA cm^−2^ just below 2 V in the sun. This work provides ideas for the preparation of a hierarchical hollow structure OER catalyst in electrolytic seawater.

## 2. Results and Discussion

### 2.1. Preparation and Characterization

**Figure 1:**
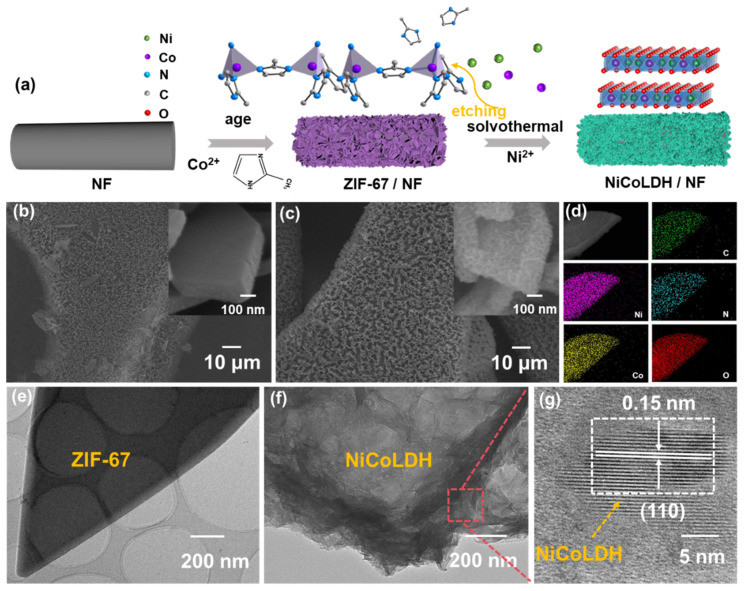
(**a**) The preparation process of NiCoLDH/NF, (**b**) SEM images of ZIF-67/NF, and (**c**) NiCoLDH/NF. (**d**) EDS elemental mappings of NiCoLDH/NF, (**e**) TEM images of ZIF-67/NF, (**f**) TEM, and (**g**) HR-TEM images of NiCoLDH/NF.

The synthesis strategy of NiCoLDH/NF is shown in Figure 1a. ZIF-67 was first grown on nickel foam directly, and then Ni^2+^ was added to hydrolytically etch the Co-N bond in ZIF-67 because of different redox potentials of nickel and cobalt [16]. Co^2+^ from ZIF-67 and Ni^2+^ from nitrate were redeposited together to form NiCoLDH nanosheets on the framework of ZIF-67. The amount of Ni^2+^ and the actual atoms used in this study are shown in Table S1 and Figure S1, respectively.

Scanning electron microscopy images (SEM, Figure 1b) show that the morphology of obtained ZIF-67 comprises a smooth nanosheet structure with an average thickness of 160 nm. As seen from Figure 1c, LDH flakes can grow in situ on a ZIF-67 template. With the Ni^2+^ content increasing, ZIF-67 nanosheets gradually transform into hollow structures composed of smaller LDH nanofragments (Figure S2). In total, 0.025 M Ni^2+^ is proved to be the best degree of ion etching, in which the hollow nanosheets are uniformly distributed on the surface of nickel foam, just like seaweeds (the Ni/Co atomic ratio is 2.21). The energy-dispersive X-ray spectroscopy (EDS) elemental mapping (Figure 1d) unequivocally shows the uniform distribution of Ni, Co, C, N, and O across the entire sheets. The transmission electron microscope (TEM) images in Figure 1e,f clearly show that ZIF-67 has a smooth surface. However, after Ni^2+^ etching, the polyhedron structure presents a hollow structure assembled by ultrathin nanosheets. These folded nanosheets grow on the surface of the ZIF-67 template, making their structure loose and uniform, exposing more active sites for the rapid diffusion of ions. From the high-resolution transmission electron microscope (HR-TEM) image in Figure 1g, clear lattice stripes with lattice spacing of 0.15 nm that come from the (110) plane of NiCoLDH (PDF#33-0429) can be seen. These results strongly suggest that the hollow NiCoLDH structure was successfully fabricated.

**Figure 2:**
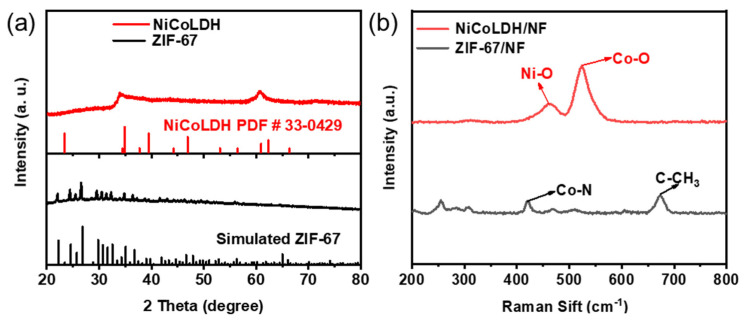
(**a**) XRD and (**b**) Raman patterns of NiCoLDH and ZIF-67.

X-ray diffraction (XRD) was used to obtain an image of the crystal structure. As shown in Figure 2a, the diffraction peaks of ZIF-67 at 22, 24.4, 25.5, and 26.6° correspond to the (114), (233), (224), and (134) planes, respectively, which is consistent with the simulated results of ZIF-67 [17]. After Ni^2+^ etching, the diffraction peaks of ZIF-67 disappear, and new peaks occur. The new diffraction peaks at 34° and 60.8° align with the (101) and (110) planes of NiCoLDH (PDF#33-0429). For a more in-depth examination of the structures of ZIF-67/NF and NiCoLDH/NF, Raman spectra were obtained. As shown in Figure 2b, the peak at 415 cm^−1^ is attributed to the Co-N stretching mode in ZIF-67, and the peak at 675.6 cm^−1^ is associated with the vibrational mode of 2-methylimidazolate (MIM) [18]. Similar to XRD results, after Ni^2+^ etching, the vibrational peaks of ZIF-67 disappear. Peaks at 462.6 cm^−1^ and 524 cm^−1^ correspond to the Ni-O and Co-O stretching of NiCoLDH, respectively [19]. Both XRD and Raman spectra prove that ZIF-67 was completely transformed into NiCoLDH.

**Figure 3:**
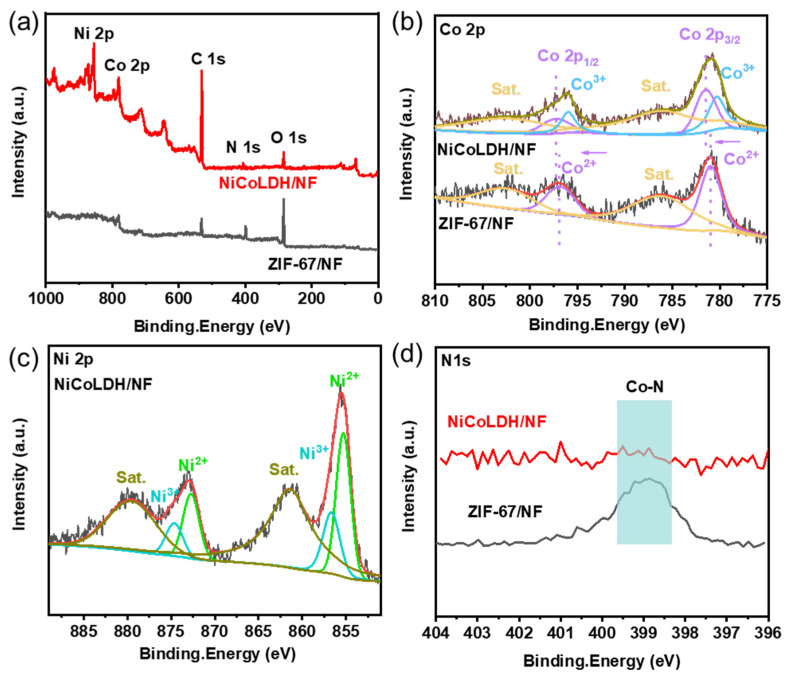
High-resolution XPS spectra of NiCoLDH/NF and ZIF-67/NF. (**a**) Overall survey of pristine; (**b**) Co 2p; (**c**) Ni 2p; (**d**) N 1s.

X-ray photoelectron spectroscopy (XPS) was employed to investigate the alterations in the surface chemical states of ZIF-67/NF and NiCoLDH/NF. The full XPS spectrum of NiCoLDH/NF in Figure 3a demonstrates the existence of Ni, Co, N, C, and O in NiCoLDH, corresponding with the EDS mapping. As seen from the high-resolution Co 2p spectra in Figure 3b, two distinct sets of peaks corresponding to Co 2p_3/2_ and Co 2p_1/2_, along with two satellite peaks, are observed in both ZIF-67/NF and NiCoLDH/NF. Compared with the peaks in ZIF-67/NF, the peaks of Co^2+^ shift toward the higher bond energy direction in NiCoLDH/NF. The enlarged binding energy of Co^2+^ is linked to the addition of Ni^2+^, which optimizes the local electronic structure of the catalytic active center [20,21]. Moreover, new peaks (780.3 and 796 eV) attributed to Co^3+^ occur, which is ascribed to the generation of NiCoLDH [22]. In the high-resolution Ni 2p spectra (Figure 3c), the typical peaks of Ni 2p_3/2_ and Ni 2p_1/2_ for Ni^2+^ appear clearly [23]. The presence of Ni^3+^ is due to the oxidation of oxygen and nitrate under reaction conditions, further confirming the generation of NiCoLDH [24]. In Figure 3d, the N 1s spectrum of ZIF-67/NF shows a strong peak of pyridinic N (399 eV), whereas the intensity is reduced significantly in NiCoLDH/NF. The reduction confirms that the imidazole ring is broken and that Co bonds to the O of LDH in NiCoLDH [25,26,27]. The O1s pattern of NiCoLDH/NF (Figure S3) shows three fitted peaks at 530.2, 531.3, and 532.8 eV, which were ascribed to M-O, O-C-O, and C-OH species, respectively [28,29]. Due to the generation of NiCoLDH, the binding energy exhibited varying degrees of growth, which is in agreement with the Raman results.

### 2.2. OER Performance in Alkaline Electrolyte

The electrocatalytic OER performance is investigated by linear sweep voltammetry (LSV) with a scan rate of 1 mV s^−1^ by using a standard three-electrode system at room temperature. RuO_2_ is a widely recognized commercial catalyst for the OER and serves as a standard reference for performance evaluations.

**Figure 4:**
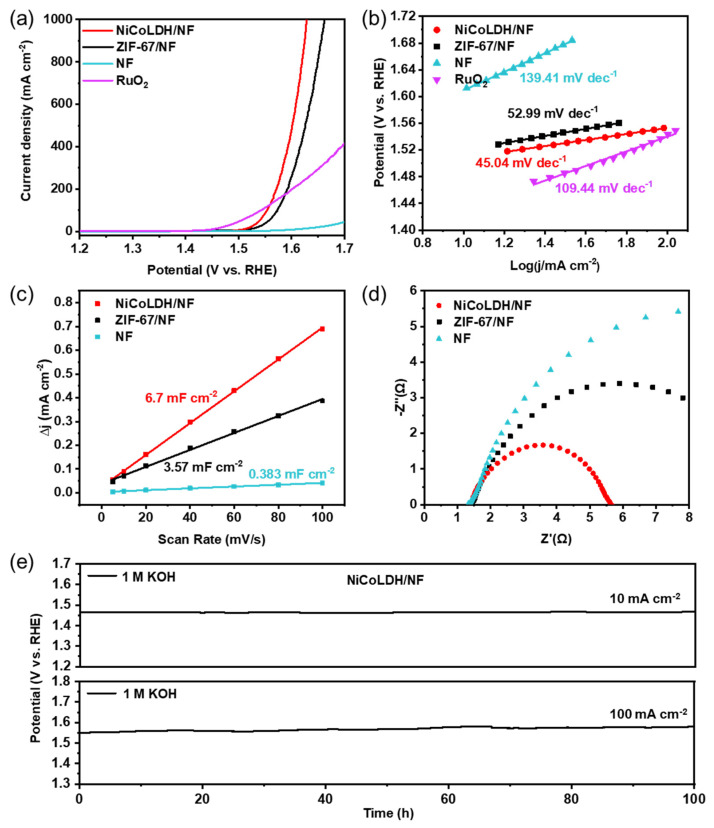
Electrocatalytic OER performance in 1.0 M KOH. (**a**) Polarization curves, (**b**) Tafel slopes, (**c**) capacitive currents versus scan rates, and (**d**) EIS Nyquist plots of NiCoLDH/NF, ZIF-67/NF, NF, and RuO_2_. (**e**) Chrono potentiometric tests of NiCoLDH/NF at constant current densities of 10 mA cm^−2^ and 100 mA cm^−2^ in 1.0 M KOH.

The performance was first detected in a 1 M KOH (pH = 14) freshwater electrolyte. As shown in Figures S4 and S5, the Ni/Co atomic ratio can seriously affect OER performance. When the Ni/Co atomic ratio is 2.21, the electrode displays the best OER performance. In the field of electrocatalysis, RuO_2_ is a widely recognized commercial catalyst for the OER and serves as a standard reference for performance evaluations. As shown in Figure 4a, at 100 mA cm^−2^, overpotential of NiCoLDH/NF is only 320 mV in freshwater, which is close to RuO_2_/NF (313 mV) electrocatalysts and better than ZIF-67/NF (344 mV). However, the Tafel slope of NiCoLDH/NF is just 45.04 mV dec^−1^, which is significantly less than that of RuO_2_ (109.44 mV dec^−1^), ZIF-67/NF (52.99 mV dec^−1^), and NF (139.41 mV dec^−1^, Figure 4b). Therefore, the OER kinetics of NiCoLDH/NF are better than those of RuO_2_, ZIF-67/NF, and NF, especially under high current density. The cyclic voltammetry (CV) curves at various scan rates and the corresponding electrochemical double-layer capacitance value (C_dl_) in 1 M KOH are shown in Figure 4c and Figure S6, respectively. The C_dl_ value of NiCoLDH/NF (6.7 mF cm^−2^) is higher than that of ZIF-67/NF (3.57 mF cm^−2^), indicating that the hierarchical hollow structure of the NiCoLDH/NF electrode can expose more active sites. To eliminate the impact of the electrochemical surface area (ECSA) for OER activity, the normalized LSV curves of ECSA are shown in Figures S7 and S8. It is clear that NiCoLDH/NF continues to demonstrate superior catalytic performance for the OER, indicating that its outstanding OER efficiency is derived not only from its extensive active area but also from its inherent catalytic properties. It can be seen from the EIS Nyquist plots (Figure 4d) that the charge transfer resistance decreases in the order of NiCoLDH/NF < ZIF-67/NF < NF, further revealing its excellent charge transfer ability. The long-term stability of the electrocatalyst is another important indicator of electrocatalyst performance. As shown in Figure 4e, at both 10 mA cm^−2^ and 100 mA cm^−2^ current densities, the OER performance of NiCoLDH/NF barely degrades after 100 h, further confirming its high catalytic activity and stability in the 1 M KOH alkaline solution. To gain a deeper insight into the superior performance of the NiCoLDH/NF catalyst, we compared it with previously reported MOF and LDH electrocatalysts in Table S2. NiCoLDH/NF significantly improves the OER electrocatalytic activity, especially at high current densities.

**Figure 5:**
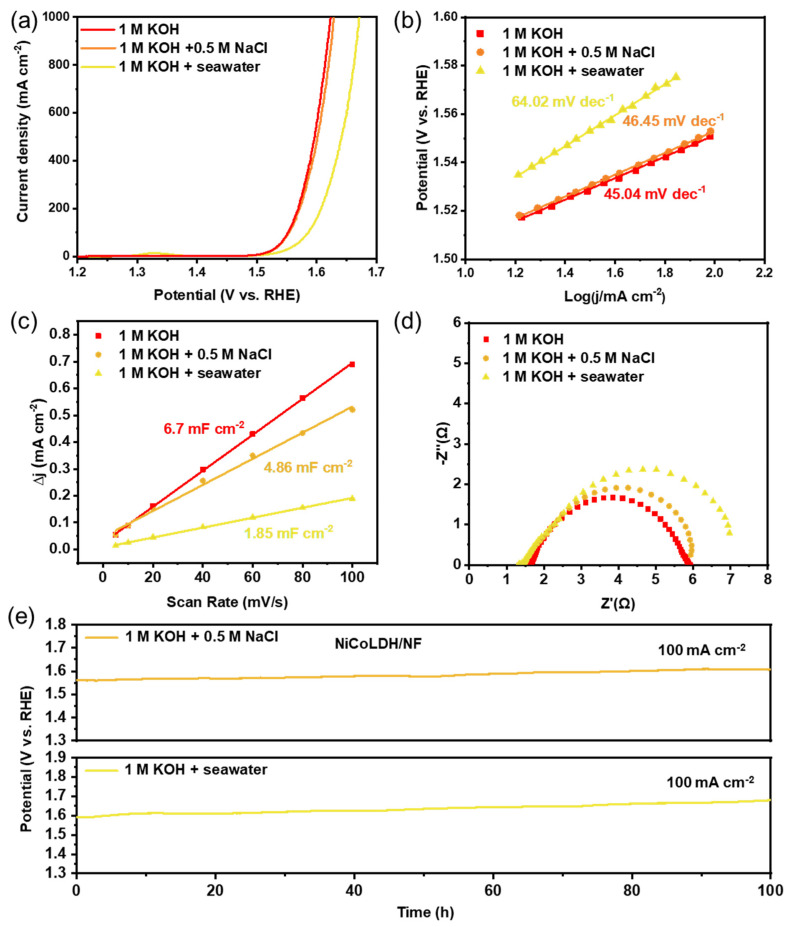
(**a**) Polarization curves, (**b**) Tafel slopes, (**c**) C_dl_, and (**d**) EIS Nyquist plots of NiCoLDH/NF in different electrolyte solutions. (**e**) Chrono potentiometric tests of NiCoLDH/NF at constant current densities of 100 mA cm^−2^ in 1.0 M KOH + 0.5 M NaCl and 1.0 M KOH + seawater.

Based on NiCoLDH/NF’s excellent OER performance in freshwater, we further tested its catalytic performance in seawater. Figure 5a shows the OER performance of NiCoLDH/NF in alkaline simulated seawater (1M KOH + 0.5M NaCl, pH = 14) and natural seawater (1M KOH + seawater, pH = 14). Natural seawater was taken from Xiaomai Island, Qingdao, China. Its OER performance in simulated seawater (322 mV at 100 mA cm^−2^) was similar to that in freshwater (Figure 5a). In addition, the Tafel slopes (64.02 mV dec^−1^, Figure 5b), C_dl_ (4.86 mF cm^−2^, Figure 5c), and EIS (Figure 5d) in simulated seawater are similar to those in freshwater, indicating that chloride ions in electrolysis cannot affect the reaction kinetics and the exposure of active sites on the catalyst. We further studied NiCoLDH/NF’s catalytic performance in natural seawater. As shown in Figure 5a, the overpotential was just 355 mV at 100 mA cm^−2^. An overpotential of far less than 480 mV was required to trigger CER (2Cl^−^ + 2OH^−^ → OCl^−^ + H_2_O + e^−^). In order to investigate whether active chlorine is generated in the OER process, we analyzed the solutions after the OER. If active chlorine (OCl^−^) is generated, OCl^-^ can react with 0.5 M KI, resulting in a color change of the solution [30]. As shown in Figure S9, the color of the NaCl solution after the OER test changes after adding KI, but there is no color change in the alkaline simulation and natural seawater, indicating that NiCoLDH/NF can meet the demands of seawater electrolysis. The Tafel slope was 64.02 mV dec^−1^ (Figure 5b) and the C_dl_ was 1.85 mF cm^−2^ (Figure 5c and Figure S10). Although the OER performance in natural seawater deteriorated slightly compared with that in simulated seawater, this was already an exciting result (Table S3). The decline in catalytic performance was mainly ascribed to the metal ions (Ca^2+^, Mg^2+^) in the natural seawater solution that may have covered and affected the active sites on the electrode surface [30,31]. EIS Nyquist plots of NiCoLDH/NF in natural seawater were larger than others (Figure 5d). The durability of long-term operations with high current densities is particularly important. As shown in Figure 5e, in simulated seawater, the overpotential almost does not increase at a current density of 100 mA cm^−2^ after 100 h of operation. Indeed, in the stability test of natural seawater, there is a little increase in overpotential. It can be seen that the sediments in natural seawater can mask the electrode surface during the testing process (Figures S11 and S12), resulting in unsatisfactory performance. As this phenomenon is common, simultaneous magnetic stirring may be an effective way to prevent sediments from covering the electrode surface.

**Figure 6:**
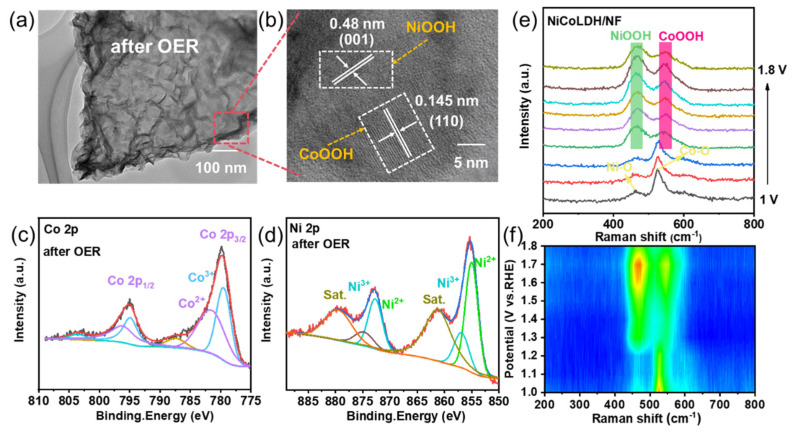
(**a**) TEM, (**b**) HR-TEM, (**c**) Co 2p, and (**d**) Ni 2p of used NiCoLDH/NF after OER in 1 M KOH + 0.5 M NaCl. (**e**) In situ Raman spectra and (**f**) the corresponding contour plot of NiCoLDH/NF under different applied potentials.

Due to the complex environment in natural seawater, the composition of the electrode surface is complex after a long-term OER, which is not conducive to an accurate analysis (Figure S10). Therefore, we used the electrode after 24 h of stability testing in simulated seawater to study the catalytic mechanism. As depicted in Figure 6a, the morphology of hollow nanosheets remains basically unchanged. In HR-TEM, lattice parameters of 0.48 and 0.145 nm are assigned to the (001) plane of NiOOH (nickel oxyhydroxide) and the (110) plane of CoOOH (cobalt oxyhydroxide) (Figure 6b), respectively. In addition, in the XPS spectra of Co2p (Figure 6c) and Ni 2p (Figure 6d), the peaks of Co^2+^ and Co^3+^ move towards the low binding energy, and the peaks of Ni^3+^move towards the high binding energy, indicating that Ni and Co substances are further oxidized with the emergence of NiOOH and CoOOH species in the OER process. Subsequently, the in situ Raman technique was used to further detect the change of NiCoLDH’s structure during the OER process. As shown in Figure 6e,f, with the increase in applied voltage (1–1.8 V), the Ni-O and Co-O bonds belonging to NiCoLDH gradually transform into NiOOH and CoOOH, further suggesting that metal-O is converted to metal-OOH in the OER process [32,33,34]. Ni cooperates with Co to participate in the catalytic process, improving the intrinsic activity of the catalyst.

### 2.3. Practical Performance in Overall Water Splitting

**Figure 7:**
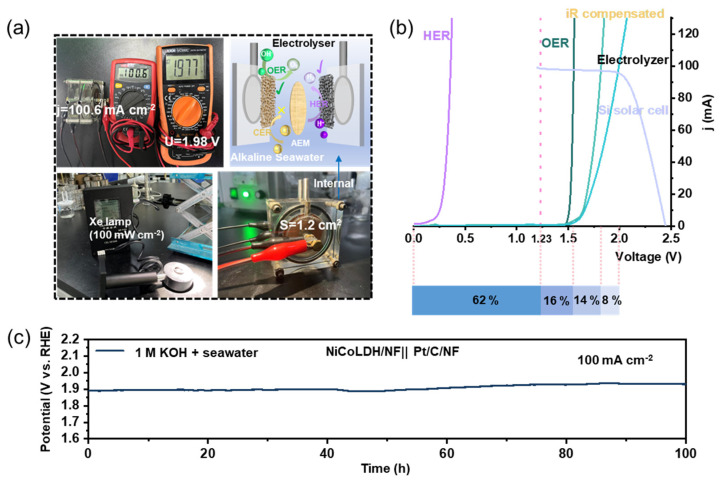
(**a**) Photographs of the electrolyzer operating in seawater using a commercial polycrystalline Si solar cell under AM 1.5 conditions (100 mW cm^−2^). (**b**) Polarization curves and the energy utilization of the electrolysis cell in seawater. (**c**) Chrono potentiometric tests of NiCoLDH/NF ||Pt/C/NF at 100 mA cm^−2^ in 1.0 M KOH + seawater.

Considering the excellent OER performance of the NiCoLDH/NF catalyst, we assembled a dual-electrode alkaline electrolytic cell in this work by using NiCoLDH/NF as the anode and commercial Pt/C/NF as the cathode to further investigate the overall seawater splitting performance. As shown in Figure 7a, driven by a silicon solar panel, the current density of the dual-electrode electrolytic cell can reach 100.4 mA cm^−2^ below 2 V in natural seawater when the sun’s radiation value is at 100 mW cm^−2^. Accurate measurements performed using the Gamry electrochemical workstation reveal that the voltage of the electrolyzer needed to generate a 100 mA cm^−2^ current density is just 1.82 V (Figure 7b). By calculating the proportion of energy consumption under practical operating conditions, the efficiency loss of the OER is only 16%, and the overall electrolysis efficiency is about 62% at a high current of 100 mA cm^−2^. Durability performance is another very crucial metric for assessing catalytic performance in practice. As shown in Figure 7c, during the 100 h test in alkaline natural seawater, the voltages increase no more than 10% at 100 mA cm^−2^, verifying the superior durability of the catalyst.

## 3. Conclusions

In conclusion, we prepared one kind of bimetallic hierarchical hollow nanosheets via Ni^2+^ etching. The experiments proved that Ni^2+^ modulates the local electronic structure of the catalytic metal site. The insertion of LDH promotes the breakage of the imidazole ring and enhances the strength of the Co-O bond, which results in an increase in the valence states of Co and Ni. With regard to benefitting from the hollow structure, not only does the transport of ions improve significantly but so does the catalytic activity area, which increases greatly. At a current density of 100 mA cm^−2^, the overpotential is 320 mV in 1 M KOH and 355 mV in alkaline seawater. The assembled dual-electrode electrolytic cell works stably for over 100 h at 100 mA cm^−2^ in natural seawater electrolytes. In situ Raman spectroscopy clearly exhibits changes in chemical bonds during the OER process, revealing the active metal-OOH intermediates.

## Data Availability

The data that support the findings of this study are available on request from the corresponding author Guoling Li.

## Supplementary Materials

The following supporting information can be downloaded at https://www.mdpi.com/article/10.3390/ma17102298/s1. A further elaboration on experimental materials and methods; figures displaying reference electrode calibrations, SEM, TEM, electrochemical analyses and measurements, LSV plots, CV curves, and integral calculations; tables presenting a performance comparison with previous studies.
