# Supplementary material for: Nickel–Cobalt Bimetal Hierarchical Hollow Nanosheets for Efficient Oxygen Evolution in Seawater
Source: Materials (Basel). 2024 May 13;17(10):2298. doi: 10.3390/ma17102298 (PMC11123210; doi:10.3390/ma17102298)
Supplement: Supplementary file 1 [file materials-17-02298-s001.zip › materials-2976414-supplementary.pdf]

Supporting Information

1.Experimental section

1.1 Chemicals Used

Ni-foam (NF) (99.9%, Sigma-Aldrich), nickel nitrate ($Ni(NO_3)_2 \cdot 6H_2O$, 99%, Macklin), cobaltous nitrate ($Co(NO_3)_2 \cdot 6H_2O$, 99%, Macklin), 2-Methylimidazole ($C_4H_6N_2$, 99%, Macklin), Ethanol (CH_3CH_2OH, Fuyu Fine Chemical), Ruthenium (IV) oxide (RuO_2,99.9%, Adamas), Platinum on graphitized carbon (Pt/C, 5 wt%, Macklin) were used as received.

1.2 Synthesis of ZIF-67/NF

Solution A: 0.291 g Co $(NO_3)_2 \cdot 6H_2O$ and 20 ml of deionized water were mixed by sonication. Solution B: Mix 0.6568 g of 2-Methylimidazole and 20 ml of deionized water by sonication. Solution B was quickly poured into solution A and 4 pieces of nickel foam were added and allowed to stand for 3 hours at room temperature. Rinse several times with deionized water and ethanol, then dry overnight under vacuum.

1.3 Synthesis of NiCoLDH/NF

Ni $(NO_3)_2 \cdot 6H_2O$ and anhydrous ethanol were mixed well and decanted into a Polytetra fluoroethylene lining. Then, ZIF-67/NF was added and the reaction was solvothermal reacted at 90 °C for 2 hours. The concentration of Ni^{2+} can be 0.00625, 0.0125, 0.025 and 0.0375 M. Rinse several times with deionized water and ethanol, then dry overnig ht under vacuum. Samples with different atomic ratio (Ni/Co) were named NiCoLDH/ NF-1.24, NiCoLDH/NF-1.47, NiCoLDH/NF-2.21 and NiCoLDH/NF-2.69.

1.4 Material characterization

The products are characterized by X-ray diffraction (XRD, Rigaku Ultima IV X-ray diffractometer with Cu Kα), Raman Spectroscopy (Renishaw in Via Plus with laser at 532 nm), Scanning Electron Microscopy (SEM, JEOL Model JSM-7800F), High-resolution Transmission Electron Microscopy (TEM, JEOL Model JSM-2100 Plus). Elemental binding energy measurements were carried out by X-ray photoelectron spectroscopy (XPS, PHI5000 Versaprobe III). The excitation source was monochromatized Al Kα radiation (hν = 1486.6eV) operated at 25W. The size of

analyzed area was 100 μm (spot diameter) and the electron emission angle was 90◦. The sample was tested directly without sputter- etched. During spectra acquisition, the base pressure was 1×10^{-8}Pa.

1.5 Electrochemical measurements

The samples synthesized on the nickel foam were directly used for electrocatalytic OER. All electrochemical measurements of the sample were carried out at room temperature. The data is recorded using a Gamry electrochemical workstation (interface1010, US) in a standard three-electrode setup. Nickel foam (NF) loaded sample (1×1 cm^2), Platinum tablet (1×1 cm^2) and Hg/HgO electrode were used as working electrode, counter electrode and reference electrode, respectively. All reported potentials are converted to reversible hydrogen electrode (RHE) scale in our work, OER is measured at a scanning rate of 1 mV s^{-1}, the electrolyte is 1 M KOH, the potentials presented in this work were referenced to the reversible hydrogen electrode (RHE) through RHE calibration, E(RHE) = E(Hg/HgO) + 0.241 V + (0.059 × pH) V. The Tafel slopes were calculated by plotting the overpotential against log |j| from LSV data. Before the LSV measurements, the catalysts was measured after 50 cycles of cyclic voltammetry (CV) to reaching a stable state. All polarization curves were corrected by the 85% iR drop compensation.

The electrochemical impedance spectroscopy (EIS) was collected at the potential corresponded to the current at 10 mA cm^{-2} with an AC amplitude of 5 mV in a frequency range from 100 kHz to 0.01 Hz. The impedance spectra were fitted using an equivalent RC circuit model. The charge transfer resistance (Rct) was then determined from the diameter of the semicircle in the Nyquist plots. The electrochemically active surface areas of the catalysts are compared on a relative scale using the capacitance of the electrochemical double layer (Cdl) at the non-Faradaic region. The electrochemical surface area (ECSA) was determined using the formula ECSA = Cdl/Cs, with Cs set at 40 μF.cm^{-2} based on the reported value for the flat electrode in a 1.0 M KOH aqueous electrolyte.

The electrocatalytic OER performance is investigated by linear sweep voltammetry (LSV) with a scan rate of 1 mV s^{-1} in 1.0 M KOH and seawater freshwater

electrolyte, by using a standard three- electrode system at room temperature. For comparison, RuO_2/NF, ZIF-67/NF and Ni foam (NF) were also evaluated with the same comparison criteria.

1.6 Solar-driven water electrolysis

The overall water splitting tests were carried out in a self-made two-electrode system in 1 M KOH using NiCoLDH/NF and Pt/C/NF as anode and cathode, respectively. The dual electrode system is composed of commercial polysilicon solar cells (5.5 × 5.5 cm^2) driven by Xe lamp as a light source under AM 1.5. The polarization curve was obtained at a scan rate of 1 mV s^{-1}. For long-term stability measurements, chronopotentiometric V-t profile data was collected for 100 h by applying consecutive constant current densities of 100 mA cm^{-2}.

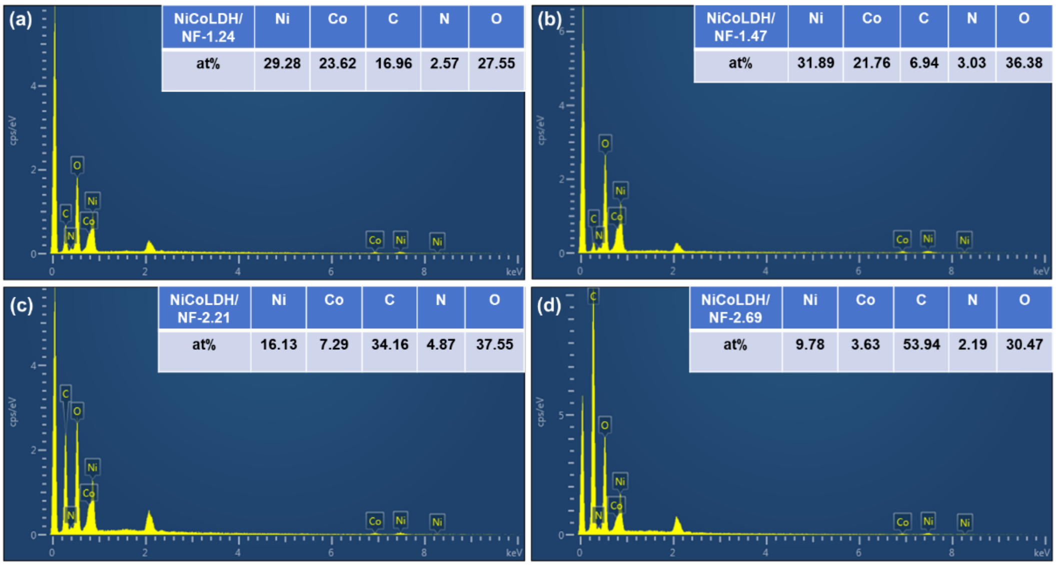

Figure S1. EDX spectrum of the NiCoLDH/NF-1.24, NiCoLDH/NF-1.47, NiCoLDH/NF-2.21 and NiCoLDH/NF-2.69. This number represents the ratio of Ni/Co

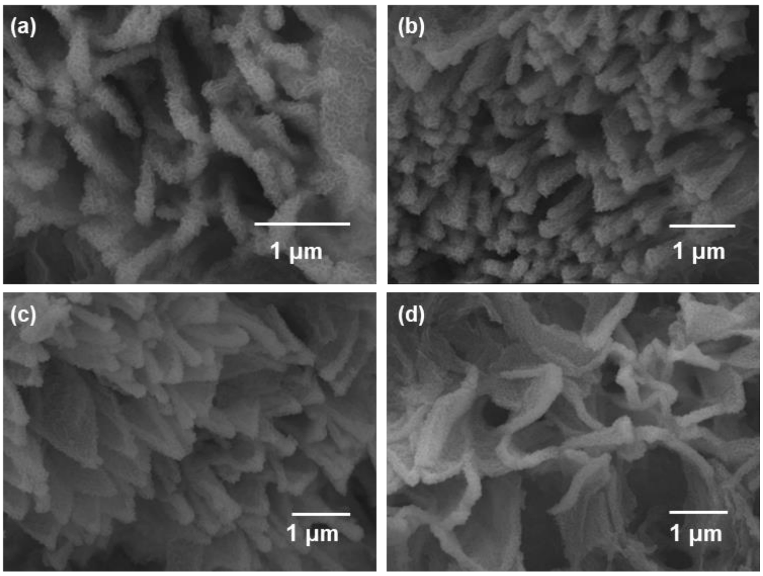

Figure S2. SEM images of the NiCoLDH/NF-1.24, NiCoLDH/NF-1.47, NiCoLDH/NF-2.21 and NiCoLDH/NF-2.69.

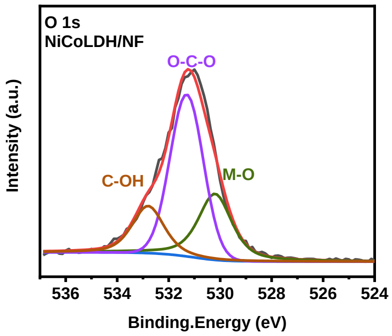

Figure S3. O 1s of NiCoLDH/NF.

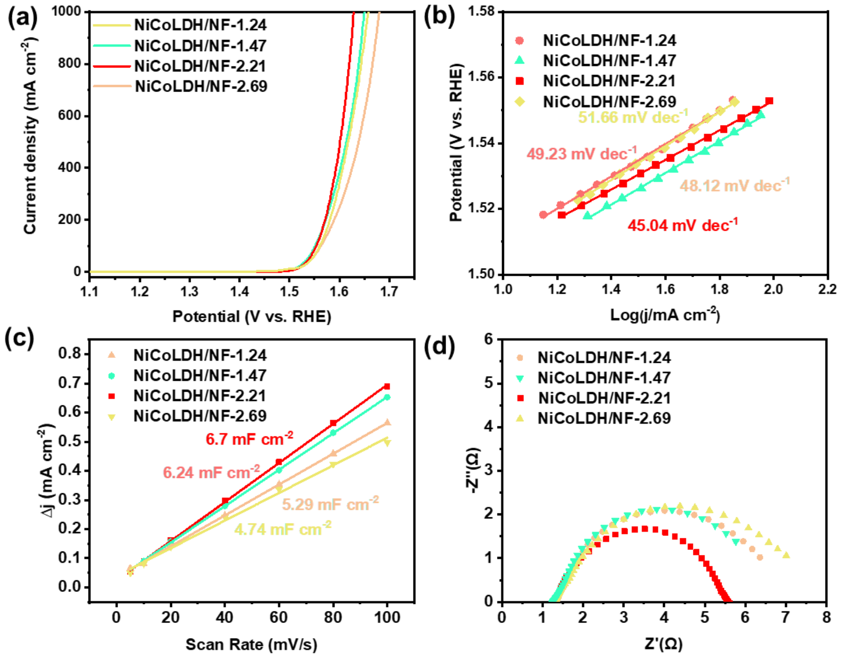

Figure S4. (a) Polarization curves, (b) Tafel slops (c) C_{dl} and (d) EIS Nyquist plots of NiCoLDH/NF-1.24, NiCoLDH/NF-1.47, NiCoLDH/NF-2.21 and NiCoLDH/NF-2.69.

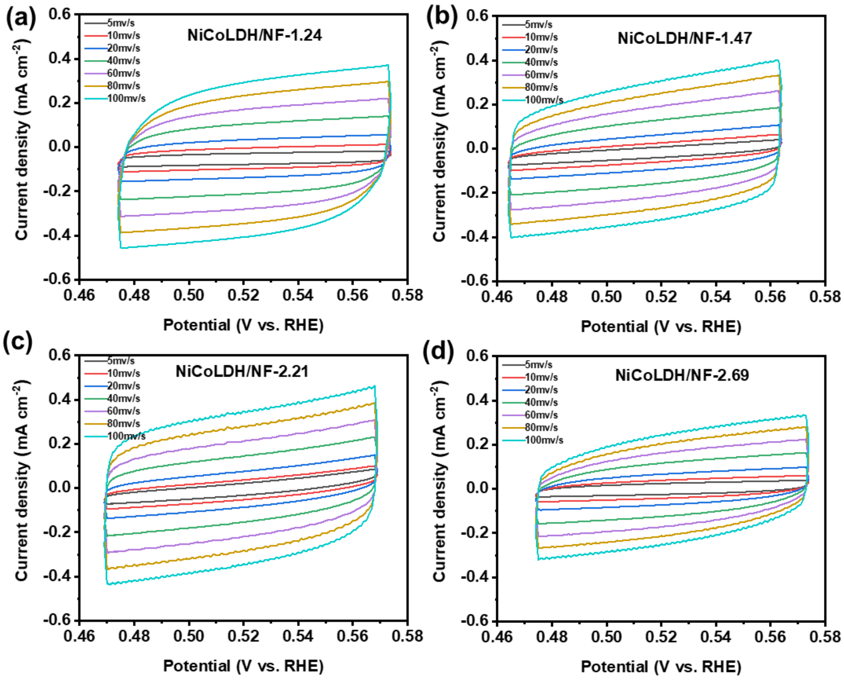

Figure S5. CV curves of NiCoLDH/NF-1.24, NiCoLDH/NF-1.47, NiCoLDH/NF-2.21and NiCoLDH/NF-2.69 samples at different scanning rates in 1.0 M KOH.

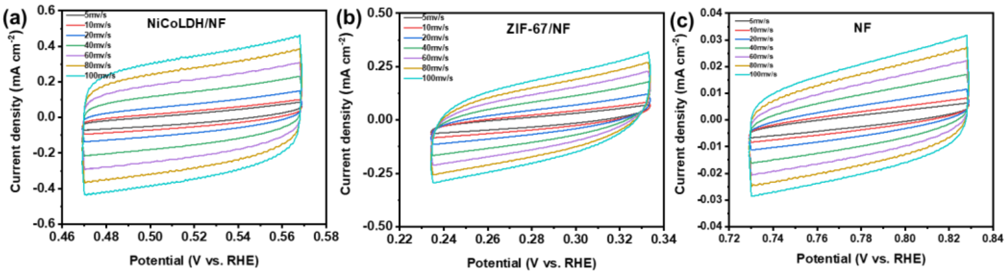

Figure S6. CV curves of NiCoLDH/NF, ZIF-67/NF and NF samples at different scanning rates in 1.0 M KOH.

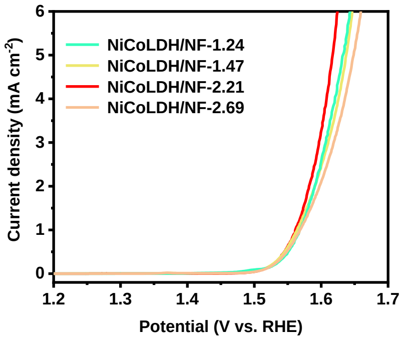

Figure S7. ECSA-normalized LSV curves of NiCoLDH/NF-1.24, NiCoLDH/NF-1.47, NiCoLDH/NF-2.21 and NiCoLDH/NF-2.69.

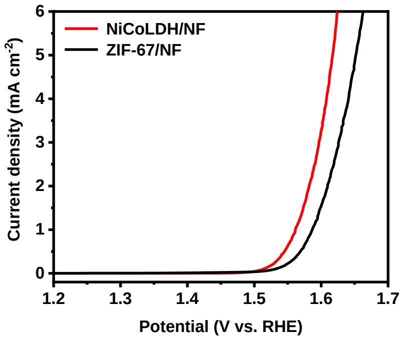

FigureS8. ECSA-normalized LSV curves of NiCoLDH/NF and ZIF-67/NF.

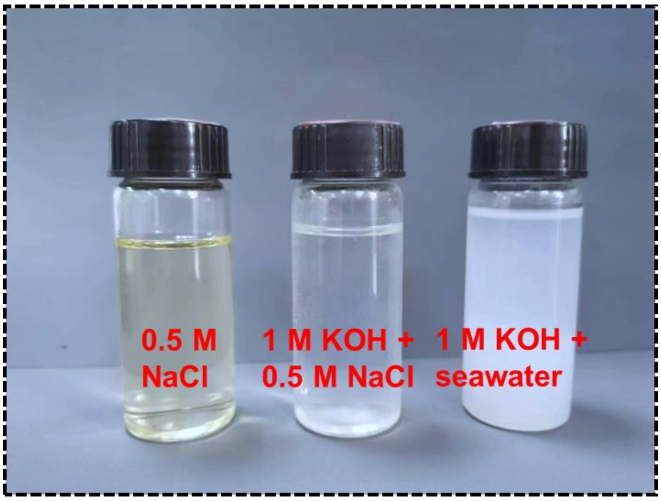

Figure S9. The color change of the solution with the addition of KI.

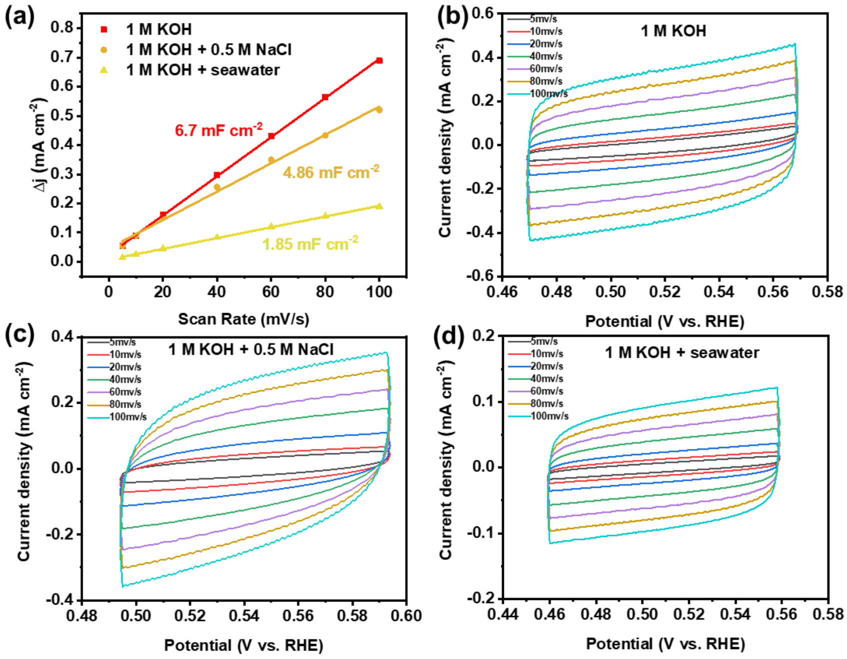

Figure S10. CV curves of NiCoLDH/NF at different scanning rates in 1.0 M KOH, 1.0 M KOH + 0.5 M NaCl and 1.0 M KOH + seawater.

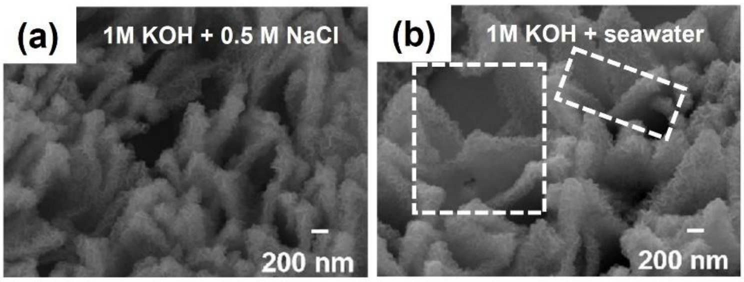

Figure S11. SEM images of the NiCoLDH/NF catalyst after testing its stability for 100

hours in 1M KOH + 0.5 M NaCl and 1.0 M KOH+ seawater.

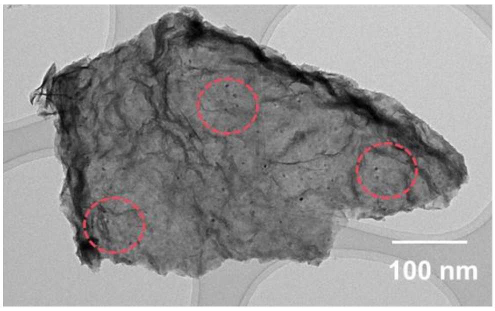

Figure S12. The TEM of NiCoLDH/NF after OER in 1.0 M KOH + seawater.

Table S1. Add different molar concentration of $Ni^{2+}$and obtain the ratio of Ni and Co through EDX spectrum.

Ni^{2+}(M)	Ni	Co	Ni/Co
6.25×10^{-3}	29.28	23.62	1.24
0.0125	31.89	21.76	1.47
0.025	16.13	7.29	2.21
0.0375	9.78	3.63	2.69

Table S2. Comparison of the electrocatalytic activity for OER in 1.0 M KOH solution with reported catalysts.

Catalyst	η(mV)at 100 mA/cm^2	Tafel slope (mV/dec)	Stability (h)	Ref.
NiCoLDH/NF	320	45.04	100	This work
CoCe-MOF/CP	363	96.1	100	1
NiCo LDH@NiFe-MIL-0.75	345	75.1	75	2
ZIF-67@CNTs	342	57	800	3
NiCoFe-HO@NiCo-	378	49.7	12	4

LDHYSMRs				
Co/NCP@NiCo-LDHs	359	81.63	20	5
NCP@WPCA	454	94.44	12	6
$CoSe_2$-30	341	54.3	25	7
$Co_3O_4/NiCo_2O_4$/NF	388	68	60	8
NiCo LDH@ZIF-67-VO/NF	348	58	18	9
NiCoLDH	407	40	6	10

Table S3. Comparison of the electrocatalytic activity for OER in alkaline simulated seawater or alkaline seawater with reported catalysts.

**Catalyst**	**ŋ(mV)at 100 mA/cm^2**	**Tafel slope (mV/dec)**	**Stability (h)**	**Electrolyte**	**Ref.**
**NiCoLDH/NF**	**322**	**46.45**	**100**	**0.5 M NaCl**	**This work**
	**355**	**64.02**	**100**	**seawater**	
CoS_2@NiFe-LDH	256	79.15	48	seawater	11
FeNiCoP@CNC	328	82.5	17	0.5 M NaCl	12
NiCoHPi-Ni_3N/NF	365	79.8	120	0.5 M NaCl	13
N-CDs/NiFeLDH	285		20	0.5 M NaCl	14
N-CDs/NiFeLDH	340		20	seawater	14
$Co_{0.4}Ni_{1.6}$P-	345	79.3	20	0.5 M NaCl	15

CeO_2/NF					
Fe_2P-NiCoP	454	49	30	seawater	16
$Ni(OH)_2$-TCNQ/GP	382	75	80	seawater	17
Fe-Co-S/Cu2O/Cu	390		30	0.5 M NaCl	18
Fe-Co-S/Cu2O/Cu	440		30	seawater	18
NiCoP@NiCo LDH/NF	420	73	50	0.5 M NaCl	19
NiCoS/NF	360	42	100	seawater	20

References

1 Y. Y. Liao, Y. Xiao, Z. Q. Li, X. Q. Zhou, J. H. Liu, F. Guo, J. Li and Y. X. Li, *small,* 2023, 2307685.

2 L. D. Yang, L. Jin, K. Wang, H. Xu, G. Y. He and H. Q. Chen, *Colloids Surf. A,* 2023, **672**, 131720.

3 H. B. Jung, Y. Kim, J. Lim, S. Cho, M. Seo, I. S. Kim, M. Kim, C. Lee, Y. W. Lee, C. Y. Yoo, Y. Oh, J. Hong, H. S. Cho and Y. Cho, *Electrochim. Acta,* 2023, **439**, 141593.

4 Q. Niu, M. Yang, D. Y. Luan, N. W. Li, L. Yu and X. W. Lou, *Angew. Chem. Int. Ed.,* 2022, **61**, 13049.

5 Z. D. Chen, Y. Zhang, P. G. Yang, W. Xiong, X. Z. Ren, Y. L. Li, L. L. Wang, S. H. Ye, J. H. Liu and Q. L. Zhang, *J. Alloys Compd.,* 2022, **890**, 161805.

6 C. Cui, X. X. Lai, R. H. Guo, E. H. Ren, W. F. Qin, L. Liu, M. Zhou and H. Y. Xiao, *Electrochim. Acta,* 2021, **393**, 139076.

7 Z. Li, Z. Z. Jiang, W. Y. Zhu, C. C. He, P. Wang, X. Wang, T. X. Li and L. Tian, *Appl. Surf. Sci.,* 2020, **504**, 144368.

8 M. Yang, W. Lu, R. X. Jin, X. C. Liu, S. Y. Song and Y. Xing, *ACS Sustain. Chem. Eng.,* 2019, **7**, 12214-12221.

9 W. X. Chen, Y. W. Zhang, R. Huang, Y. M. Zhou, Y. J. Wu, Y. J. Hu, K. Ostrikov, *J. Mater. Chem. A,* 2019, **7**, 4950-4959.

10 H. F Liang, F. Meng, M. Cabán-Acevedo, L. S. Li, A. Forticaux, L. C. Xiu, Z. C. Wang, S. Jin, *NanoLett.,* 2015, **15**, 1421-1427.

11 X. F. Zhang, Z. X. Li, Z. W. Cai, J. Li, L. C. Zhang, D. D. Zheng, Y. S. Luo, S. J. Sun, Q. Liu, B. Tang, Y. C. Yang, H. Q. Wang and X. P. Sun, *Chem. Commun.,* 2023, **59**, 11244.

12 H. Xu, L. Jin, K. Wang, L. D. Yang, G. Y. He and H. Q. Chen, *Int. J. Hydrogen Energy,* 2023, **48**, 38324-38334.

13 P. Ding, H. Song, J. Chang and S. Lu, *Nano Res.,* 2022, 1**5**, 7063-7070.

14 Y. K. Cong, X. N. Chen, Y. Mei, J. Ye and T. T. Li, *Dalton Trans.,* 2022, **51**, 2923.

15 M. Xiao, C. Zhang, P. Wang, W. Zeng, J. Zhu, Y. Li, W. Peng, Q. Liu, H. Xu, Y. Zhao, H. Li, L. Chen, J. Yu and S. Mu, *Mater. Today Phys.,* 2022, **24**, 100684.

16 H. Sun, J. Sun, Y. Song, Y. Zhang, Y. Qiu, M. Sun, X. Tian, C. Li, Z. Lv and L. Zhang, *ACS Appl. Mater. Interfaces,* 2022, **14**, 22061–22070.

17 L. Zhang, J. Wang, P. Liu, J. Liang, Y. Luo, G. Cui, B. Tang, Q. Liu, X. Yan, H. Hao, M. Liu, R. Gao and X. Sun, *Nano Res.,* 2022, **15**, 6084–6090.

18 J. Sun, P. Song, H. Zhou, L. Lang, X. Shen, Y. Liu, X. Cheng, X. Fu and G. Zhu, *Appl. Surf. Sci.,* 2021, **567**, 150757.

19 Y. H. Wu, Z. N. Tian, S. F. Yuan, Z. Y. Qi, Y. R. Feng, Y. F. Wang, R. Huang, Y. L. Zhao, J. H. Sun, W. Y. Guo and J. L. Feng, *Chem. Eng. J.,* 2021, **411**,128538

20 C. Z. Wang, M. Z. Zhu, Z. Y. Cao, P. Zhu, Y. Q. Cao, X. Y. Xu, C. X. Xu and Z. Y. Yin, *Appl. Catal. B-Environ.,* 2021, **291**, 120071.
